# Effect of agricultural land-use change on ant dominance hierarchy and food preferences in a temperate oak forest

**DOI:** 10.7717/peerj.6255

**Published:** 2019-01-14

**Authors:** Citlalli Castillo-Guevara, Mariana Cuautle, Carlos Lara, Brenda Juárez-Juárez

**Affiliations:** 1 Centro de Investigación en Ciencias Biológicas, Universidad Autónoma de Tlaxcala, Tlaxcala, Mexico; 2 Departamento de Ciencias Químico-Biológicas, Universidad de las América Puebla, Puebla, Mexico; 3 Maestría en Biotecnología y Manejo de Recursos Naturales, Centro de Investigación en Ciencias Biológicas, Universidad Autónoma de Tlaxcala, Tlaxcala, Mexico

**Keywords:** Foraging preferences, Discovery-dominance trade-off, Functional groups, Ant communities, Numerical dominance, Mexico

## Abstract

**Background:**

The discovery-dominance trade-off is the inverse relationship between the ability of a species to discover resources and the species’ dominance of those resources; a paradigm used to explain species coexistence in ant communities dependent on similar resources. However, factors such as stress (e.g., temperature) or disturbance (e.g., removal of biomass) associated with the change in land use, can modify this trade-off. Here, we aimed to determine the potential effects of land use change on dominance hierarchy, food preferences and on the discovery-dominance trade-off.

**Methods:**

An experiment with baits was used to investigate the dominance hierarchies of ant communities in a temperate mountain habitat in central Mexico. We evaluated the dominance index (DI), food preferences and discovery-dominance trade-offs of ants inhabiting two types of vegetation: a native oak forest and agricultural land resulting from agricultural land use and grazing.

**Results:**

The ant communities in both environments were comprised of three species of ants (*Monomorium minimum, Myrmica mexicana*, and *Camponotus picipes pilosulus*), four morphospecies (*Pheidole* sp.1 and *Pheidole* sp.2,* Temnothorax* sp. and* Lasius* sp.) and one genus (*Formica* spp.). All Formicidae showed values of intermediate to low DI, and this factor did not seem to be influenced by the change in land use. Ants in the modified vegetation (i.e., agricultural land) were found to be numerically greater. Overall, a higher number of visits were registered to the tuna bait, although the duration of foraging events to the honey baits was longer. However, foraging times were dependent on the species considered: the generalized Myrmicinae, *M. minimum*, the ant species with highest DI, foraged for longer periods of time in the agricultural land and on the tuna bait. Meanwhile, the cold-climate specialist *Formica* spp., with a lower DI, foraged for longer periods of time in the oak (although not significant) and on the honey bait. We found little evidence of the discovery-dominance trade-off; instead, we found considerable diversity in the strategies used by the different species to access resources. This range of strategies is well represented by the generalized Myrmicinae *M. minimum*, the cold-climate specialists *Formica* spp. and *Temnothorax* sp., and the rare species, as the cold climate specialist *Lasius* sp. (insinuators).

**Conclusions:**

Our evaluation shows that transformation of the original habitat does not appear to affect the hierarchical dominance of the ant communities, but it does affect their food preferences. Species with higher DI values such as the generalized Myrmicinae are more skilled at resource acquisition in modified habitats. Our results suggest that change in land use promotes an increase in the diversity of foraging strategies used by different ant species. This diversity may contribute to resource partitioning which favors coexistence.

## Introduction

It has been proposed that competition is one of the mechanisms which structure animal communities that share resources, and this is also true for ant communities. However, there is currently a contentious debate about the exact role of competition on the structure of ant communities and ant dominance hierarchies (Arnan et al., 2018). Evidence has shown that ant species exert an influence on other ant species in the vicinity of their nests and that they compete for high-value food resources, but the effects of this competition at the community level are unclear (Soares, 2013; Arnan et al., 2018). Also, factors such as high temperatures, invasive species, parasitoids, and dietary differentiation have been observed to alter the role of competition on the structuring of ant communities, thereby facilitating the coexistence of ants with different competitive abilities (Parr & Gibb, 2012; Cerdá, Arnan & Retana, 2013; Houadria et al., 2015).

Interspecific trade-offs are considered to be one of the most prevalent mechanisms that enable coexistence at a local scale (Kneital & Chase, 2004). Of these, the discovery-dominance trade-off (an inverse relationship between a species’ ability to find and control resources), which was first reported by Fellers (1987), has been typically used to explain coexistence and competition in ant communities (Parr & Gibb, 2012). This relationship predicts that when dietary difference is low in an ant community, a trade-off between discovery and competitive dominance can permit coexistence. The trade-off indicates that dominant ants are not as efficient at discovering food resources, nor are subordinant ants able to dominate them (Fellers, 1987). Several studies have addressed the importance of the discovery-dominance trade-off as a structuring mechanism in ant communities, with positive (e.g., Fellers, 1987; Davidson, 1998; Delsinne, Roisin & Leponce, 2007; Pearce-Duvet & Feener Jr, 2010), or neutral effects (Wiesher, Pearce-Duvet & Feener Jr, 2011; Parr & Gibb, 2012). Other studies have found that its effect can be mediated by factors such as temperature and habitat structure (Wiesher, Pearce-Duvet & Feener Jr, 2011; Parr & Gibb, 2012).

As is the case with many other taxa, the most common use of dominance hierarchies is in the formal testing of trade-offs to explain species coexistence within ant communities. Dominance hierarchies constructed for ant communities, rank ant species in two or three levels, based on their numerical or behavioral dominance (Stuble et al., 2017). Although the number of levels and characteristics used to determine dominance hierarchies is variable (Stuble et al., 2017), factors such as the aggressiveness, territoriality, numerical prevalence and recruitment type of the species are invariably taken into account (Cerdá, Arnan & Retana, 2013). Because the ant behavioral attributes associated with hierarchical dominance such as diet or foraging time are part of their niche dimensions, these attributes can also be used to classify ants into functional groups (see Andersen, 1995). A commonly used scheme of functional groups is based on global-scale responses of ants to environmental stress (factors affecting productivity) and disturbance (factors removing biomass), operating at the genus or species-group level (Hoffman & Andersen, 2003). Andersen (1995) proposed a classification, based on seven functional groups, which is especially useful for detailed analyses of particular communities, mostly to characterize the effect of disturbance such as land use change.

Although ant hierarchy levels were first described in temperate forest species (Vepsäläinen & Pisarski, 1982), these topics have been studied more extensively in ant communities in tropical forest or arid habitats. Although data is relatively scarce, it has been found that ant communities in temperate forest are mainly structured around dominant and subordinate species (Arnan, Cerdá & Retana, 2012; Trigos Peral et al., 2016). Arnan et al. (2018), found that species richness was positively correlated with the presence of dominant native ant species, a pattern that was consistent in temperate and subtropical latitudes, because ant richness is lower in temperate forest than other habitats, dominance is expected to be lower in these communities. For example, there are up to 20 ant species in North American temperate forest (Lynch & Johnson, 1988; Cuautle, Vergara & Badano, 2016), while Mexican tropical dry forest or Australian semiarid and arid habitats may contain 70 to 100 species (Arnan, Gaucherei & Andersen, 2011; García-Martínez et al., 2015). The main factors limiting the abundance of behaviorally dominant species is stress (e.g., temperature) and disturbance (e.g., land use change). As well as modifying biomass, change of land use modifies ground temperature, especially in temperate environments (Cuautle, Vergara & Badano, 2016). These studies suggest that ant communities in temperate environments may be more sensitive to habitat disturbance and transformation (e.g., change of land use). We propose that this is either because change of land use alters the balance of competitive interactions, often in effect resetting the process of competitive exclusion, or because it clears space for colonization of new species.

Ants are widely used as bioindicators of human disturbance (Andersen & Majer, 2004), yet there is an ongoing need for research on ant responses to habitat disturbance in different parts of the world, and to determine how indicative their responses are of ecological change in general. The conservation of forest habitats is essential for species interactions and species recruitment at the local and regional scale. Here we examine the effects of change in land use on ant assemblages by comparing undisturbed oak forest and areas where the forest has undergone human-modification, i.e., agricultural land. We address three specific questions: (1) Does land-use change modify the competitive hierarchy in temperate forest ant communities? We anticipated that cold-climate specialists would dominate in the oak forest; while generalized Myrmicinae were expected to dominate the more open modified habitat (agricultural land). (2) Does the change in land use alter food resource needs/preference in these ant communities? As human-induced disturbance and transformation of habitat may alter the availability of resources (e.g., quantity or quality), we expected that ant food preferences (for specific baits) would be different between oak forest and agricultural land. (3) Does change in land use alter foraging strategies, favoring one species over another (discovery-dominance trade-off)? We expected that in the modified habitat this trade-off would be relaxed due to the absence of dominant species in disturbed habitats.

## Methods

### Study site

Ant assemblages were studied from April through September 2015 in a representative area of vegetation (∼250 ha) at the La Malinche National Park (MNP), Tlaxcala, Mexico. About half of this area is comprised of undisturbed forest with ongoing management to prevent fires. The rest is forest that has suffered numerous fire events, illegal logging, and temporal agricultural use during the last 20 years and where regrowth has been disturbed by constant grazing. The MNP forms part of the Trans-Mexican Volcanic Belt. It has an area of 46,093 ha, located between the 19°14′ N and 98°14′ W, with an altitude ranging from 2,300 to 4,461 m a.s.l. (López-Domínguez & Acosta, 2005). Mean annual precipitation is 800 mm, the rainy season is between June and October, and the mean annual temperature is 15 °C. Coniferous forest and Oak forest are the dominant kinds of vegetation.

The vegetation type present at the study site is a transition from preserved oak forest to rainfed agriculture and agricultural land used for grazing livestock, and has an elevation range between 2,900 to 2,700 m a.s.l. The forest consisted of dense stands of oak trees with 80 to 90% coverage where the dominant species are *Quercus laurina*, *Q. crassifolia*, and *Q. rugosa*. The area with a change in land use from oak forest to agricultural land includes areas without trees represented by an induced pasture mosaic, and secondary vegetation originating from the burned forest or abandoned agricultural areas. The most common species in this area are *Festuca tolucensis*, *Muhlenbergia macroura*, and *Stipa ichu*. The field research reported here was performed using the required permit (SEMARNAT No. DGVS/06901/15).

Six transects of 80 m in length were established in pairs. Each pair of transects were parallel sites of oak and agricultural land, separated by at least 500 m. Two transects were maintained at 2,900 m a.s.l., two at 2,800 m a.s.l., and two at 2,700 m a.s.l. The separation between sites was at least 1.5 km. The field experiments and ant samples carried out in each of these transects are detailed below.

### Feeding trials: dominance hierarchy, food preference, and discovery-dominance trade-off

This study employs the ant competition hierarchy created by Vepsäläinen & Pisarski (1982). Their hierarchical categorization comprises three levels: The lowest level (submissive or subordinate) consists of ant species that defend only their nest. The intermediate level (subdominant) corresponds to species that also defend the food resources they find, and the top level (dominant) consists of species that successfully defend territories that include their nests and foraging areas. These hierarchy levels correspond respectively, to the submissive, encounter and territorial categories later proposed by Savolainen & Vepsäläinen (1989). The categories take into account characteristics such as aggressiveness, territoriality, numerical prevalence and recruitment type (Cerdá, Arnan & Retana, 2013). Ants in the subordinate species category have small colonies, have simple or non-existent recruitment systems and avoid physical contact with workers of other colonies. Subdominant species are aggressive, able to defend or to take over food resources, and have moderate population densities; the dominant species are highly aggressive and numerically prevalent (Cerdá, Arnan & Retana, 2013). Both dominant types can perform group and mass recruitment.

We chose numerical dominance to assess the dominance hierarchy of the ants (Andersen, 1992; Cerdá, Retana & Cros, 1997). As numerical and behavioral dominance are highly correlated, this method has been widely accepted and used in the ant literature (Dejean & Corbara, 2003; Santini et al., 2007; Parr, 2008; Parr & Gibb, 2012; Dáttilo, Díaz-Castelazo & Rico-Gray, 2014). The method indicates which species are consistently present at the baits, and which ones dominate the baits numerically and monopolize them (Parr, 2008). It is effective when the objective is to evaluate which species of ants win the competition for a resource but does not determine how they do it.

Numerical dominance was evaluated using the numerical dominance index (DI) for each morphospecies using the formula: *N* = (D*i*)/(DI + S*i*). Here, DI is the number of baits monopolized by the species of ant *i*, and S*i* is the number of baits that the species of ant *i* used but did not monopolize. Baits were considered to be monopolized when more than five individuals (workers or soldiers) of the same morphospecies were using the resource without the presence of other morphospecies. This measure (more than five individuals) takes into consideration that in temperate climates ants are less abundant and recruitment is considered weaker than in tropical environments where the index has been used more (Santini et al., 2007). Therefore, dominant morphospecies are those that find and monopolize a larger proportion of the food resources in a given environment. The range of the index goes from 0 (completely submissive species) to 1 (totally dominant species) and is similar to the “monopolistic index” used in other studies (Fellers, 1987; Santini et al., 2007; Parr & Gibb, 2012; Dáttilo, Díaz-Castelazo & Rico-Gray, 2014).

To quantify the numerical dominance, food preference and competitive skills of each morphospecies, data were collected using an experiment with baits that was replicated six times, between April and September 2015. A sampling point was placed on every 10 m of the transects. Each sampling point consisted of paired Petri dishes (at <10 cm apart) with bait as attractants. Two types of baits were used: 0.5 ml of commercial honey diluted with 50% water and 0.5 g of commercial tuna. For each transect, there were nine sampling points with 18 Petri dishes. Each month, 108 Petri dishes were placed (2 types of vegetation × 3 transects × 9 sampling points × 2 types of bait), which adds up to a total of 648 Petri dishes. The baits were placed *ad libitum* in the center of each Petri dish. Honey and tuna are attractive not only to ants that feed on nectar but also to those that show a preference for resources rich in carbohydrates and protein (Koptur & Truong, 1998; Blüthgen & Fiedler, 2004). The observation period was from 9:00 to 17:00 h. When climatic conditions permitted, Petri dishes were placed in the first pair of transects at 9:00 h., the second pair of transects at 11:00 h., and in the third at pair at 13:00 h. The order of placement of the Petri dishes in the transects was alternated each month. Each month, the information collected during the day of sampling (108 Petri dishes) was used to calculate the numerical dominance for each species registered. The DI was calculated for each species by averaging the data collected during the 6 months of sampling.

To evaluate the discovery-dominance trade-off, observation of the attractant was carried out at three time-points of exposure: at 1 h, 2 h, and 4 h. At the outset, all Petri dishes were placed in the transects with baits. The first pair was observed continuously for an hour during which the number of ants, morphospecies, time of arrival and permanence (foraging) were quantified. The second pair of Petri dishes was exposed for two hours, but at the end of the first hour of exposure, water was added to collect the ants present in the bait and all those that arrived during the following hour. The third pair of Petri dishes was exposed for four hours, but again, at the end of the first hour of exposure, water was added to collect all the ants that arrived during the remaining 3 h. Water was added because initially, we had few visits during the first and second hour of observation, especially in the oak forest. However, we observed that if we left the Petri dish for four hours, a greater number of ants and ant species were recorded. The water did not prevent the recruitment of ants, as can be seen by a general increase in ants recorded after two and four hours. Ant activity was also observed when the Petri dishes were recovered. Although the addition of water to the bait after the first hour may have altered the interactions of species competition, it allowed us to record the continual arrival of other species to the bait during the second and fourth hrs.; that is, the ants kept coming despite the addition of water. The data obtained at the 2 and 4 h. time points were sufficient to perform the comparative analyses between times and species of ants. In addition, in the temperate environment studied, aggressive encounters between the ant species were not observed directly. On the contrary, we frequently observed more than two species foraging at the same time without inference between them. Each exposure time of the baits (1 h, 2 h, and 4 h) had three replicates, which formed the nine sampling points in each transect. This sampling allowed us to quantify differences in the ability of the ants to discover resources (based on the arrival times registered in baits exposed for 1 h) and their ability to monopolize the resource (determined by the DI of the ants found in the baits exposed for 2 h, and 4 h). All of the ants collected while visiting the baits were preserved in alcohol at 70%. Specimens of each morphospecies were assembled and identified using taxonomic keys (Mackay & Mackay, 1989) and the help of specialists from the Entomology Laboratory of the Institute of Ecology, AC and the University of Quebec in Chicoutimi, Department of Fundamental Sciences. All morphospecies identified in this study were assigned to the functional groups proposed by Andersen (1997). The reference collection was then integrated into the Entomological Collection (Formicidae) of the Entomology Laboratory of the University of Las Américas Puebla (UDLAP).

In addition to characterizing the DI of the different morphospecies of ants, their recruitment (mass or group) was determined by recording the average number of ants per Petri dish. Incidence is an indicator of the amount of resources that the different morphospecies of ants are capable of exploiting. This was calculated by observing the number of Petri dishes used by each morphospecies.

### Statistical analysis

#### Foraging preference

Pearson’s chi-square tests (with Yates correction) were performed to analyze the foraging preference of the ants, measured as the number of ants visiting each type of bait (honey and tuna) in both of the environments (oak forest and agricultural land). Also, as an alternative way of approaching the foraging preference, we used a Generalized Linear Mixed Model (GLMM) with a Poisson distribution and log link function as implemented in the *glmer* function from the *nlme* package of R (R Core Development Team, 2014). Given that longer foraging times to a bait could indicate a greater preference for that bait, this model was used to analyze variation in the duration of the foraging events. However, longer foraging times could also indicate a limitation of the natural occurrence of that resource in their environment. The full GLMM included the factors vegetation type (oak forest and agricultural land), bait type (tuna and honey), morphospecies, and their interaction. Foraging duration was treated as the dependent variable. Variation in DI values among morphospecies was determined using a GLMM with a Poisson distribution and sqrt link function in R (R Core Development Team, 2014). The full GLMM model included the following factors: vegetation type (oak forest and agricultural land), bait type (tuna and honey), bait exposure time (1 h, 2 h, and 4 h), morphospecies, and their interactions as defined above, and DI estimates treated as the dependent variable. Months (as a repeated measure) and transect were included in both models as random effects.

#### Discovery-dominance trade-off

The ant’s ability to discover the baits in the two habitats was evaluated considering the probability of occurrence of visits (ants arriving at the bait while foraging). If a morphospecies is more likely to visit a bait during the first hour of exposure and presents a high DI in this first hour, then it can be cataloged as a discoverer. On the other hand, a higher DI value at the baits exposed for 2 h and 4 h will indicate which species are potentially monopolizing resources. Species are then ranked according to its DI and Tukey test can be applied to form hierarchy groups at the different hour’s intervals.

Survival Analysis (“time failure analysis”) in R (R Core Development Team, 2014) was used to analyze differences in ant arrival times the interaction effect of vegetation type × morphospecies. Survival analysis is a branch of statistics for analyzing the expected duration of time until one or more events happen. One of the features of time failure analysis is the use of censored data. Censored data points are those in which an event is not observed because the study ended before the event could have happened to some individuals under observation. This is useful in field biology, where the observation period may be too brief for all possible events to be registered (Muenchow, 1986). The actual time of visits to the baits is not always known; only the length of time during which the event did not occur. For these data, we recorded the beginning of our observations as time zero and subsequent foraging events as minutes from start time. The Kaplan–Meier product-limit nonparametric method was used to calculate the likelihood that ants had not yet visited a bait 60 min after the start of observation, and the logrank (Mantel-Cox) statistic was used to test for differences in foraging between the types of bait and vegetation (Muenchow, 1986).

## Results

In total, 3,498 individuals were recorded during the study, which corresponded to eight morphospecies from seven genera of the subfamilies Myrmicinae (*Monomorium minimum*, *Pheidole* sp.1, *Pheidole* sp.2, *Temnothorax* sp., and *M. mexicana*) and Formicinae (*Formica* spp., *Lasius* sp., and *C. picipes pilosulus*). In the case of *Formica* spp., it was composed of three morphospecies, only *Formica retecta* was identified, and another morphospecies of the Fusca group and one more belonged to the Microgyna group. However, in the study, it was managed as a single group of *Formica* spp., as these species are functionally and competitively similar (Andersen, 1997). All morphospecies were present in both environments, except for *Pheidole* sp.2, *Lasius* sp., and *C. picipes pilosulus*, which were only recorded in the agricultural land. The highest number of individuals recorded were Myrmicinae (Table 1). Also, the number of individuals per morphospecies varied depending on the type of bait used (Table 1). These morphospecies pertain to four functional groups: generalized Myrmicinae (*Monomorium minimum*, *Pheidole* sp.1, and *Pheidole* sp.2), cold-climate specialists (*Formica* spp., *Temnothorax* sp., and *Lasius* sp.), opportunists (*M. mexicana*) and subordinate Camponotini (*C. picipes pilosulus*).

**Table 1:** Number of each ant morphospecies that arrived at the baits (tuna and honey) in both types of vegetation (oak forest and agricultural land).

	Type of vegetation	Oak forest			Agricultural land			
	Type of bait	Tuna	Honey	Total	Tuna	Honey	Total	Sum totals
FG	*Subfamily*							
	*Formicinae*							
ccs	*Formica* spp.	85	48	133	213	270	483	616
ccs	*Lasius* sp.	–	–	–	1	2	3	3
sC	*C. picipes pilosulus*	–	–	–	–	3	3	3
	*Myrmicinae*							
gM	*M. minimum*	19	7	26	1,636	337	1,973	1,999
gM	*Pheidole* sp.1	31	1	32	310	20	330	362
gM	*Pheidole* sp.2	–	–	–	236	2	238	238
ccs	*Temnothorax* sp.	42	44	86	81	96	177	263
o	*M. mexicana*	1	1	2	8	4	12	14
		178	101	279	2,485	734	3,219	3,498

**Notes.**

FG Functional Groups gM generalized Myrmicinae ccs cold-climate specialists sC subordinate Camponotini o opportunists

(−) No individuals of this morphospecies registered.

Regardless of the bait visited, the number of ants recorded was significantly higher in the agricultural land than in the oak forest (${\chi }_{\mathrm{c}}^{2}=2469.33$, *df* = 1, *P* <  0.001, oak forest *n* = 279, agricultural land *n* = 3,219). In the oak forest, a higher number of individuals were recorded of *Formica* spp. and *Temnothorax* sp., while in the agricultural land the more abundant species were *M. minimum, Formica* spp., *Temnothorax* sp., *Pheidole* sp. 1, *Pheidole* sp. 2, in that order (Table 1).

### Does the change in land use alter food resource needs/preference in these ant communities?

During the present study the total number of ants recorded in the tuna bait was higher than in the honey bait (${\chi }_{\mathrm{c}}^{2}=945.90$, *df* = 1, *P* <  0.001, tuna *n* = 2,663, honey *n* = 835) (Table 1). All morphospecies were recorded at both baits, except for *C. picipes pilosulus*, which only visited the honey bait. Significant differences in foraging preference were observed in the morphospecies *M. minimum*, *Pheidole* sp.1, and *Pheidole* sp.2. Higher numbers of these species foraged in the tuna bait (${\chi }_{\mathrm{c}}^{2}=858.47$, *df* = 1, *P* <  0.001, ${\chi }_{\mathrm{c}}^{2}=281.10$, *df* = 1, *P* <  0.001; ${\chi }_{\mathrm{c}}^{2}=228.10$, *df* = 1, *P* <  0.001, respectively). On the contrary, in *Formica* spp., *Temnothorax* sp. and *M. mexicana* the difference was minimal between the baits. (${\chi }_{\mathrm{c}}^{2}=0.58$, *df* = 1, *P* = 0.443, ${\chi }_{\mathrm{c}}^{2}=0.97$, *df* = 1, *P* = 0.3239, ${\chi }_{\mathrm{c}}^{2}=0.64$, *df* = 1, *P* = 0.4226, respectively). In the case of *Lasius* sp. the number of visits registered to either bait was too low to establish their foraging preference, and just one *C. picipes pilosulus* was recorded in the honey bait (Table 1).

The GLMM model showed significant differences in foraging duration between the ant morphospecies (regardless of the vegetation type or bait) (*χ*^2^ = 100.44, *df* = 5, *P* <  0.0001). Posthoc mean contrasts (Tukey method) showed that the foraging duration of *M. minimum* (mean ± SE = 21.14 ± 0.17 min) was significantly longer than *M. mexicana* (5.80 ± 0.27 min), *Formica* spp. (4.67 ± 0.17 min), *Temnothorax* sp. (4.50 ± 0.22 min), *Pheidole* sp.1 (3.56 ± 0.29 min) and *Lasius* sp. (*P* <  0.01). The duration of foraging events in the agricultural land site (mean ± SE = 5.95 ± 0.17 min) was longer than in the oak forest (5.40 ± 0.11 min), although the difference was not significant (*χ*^2^ = 0.386, *df* = 1, *P* = 0.535). Whereas, in both habitats, foraging events were significantly longer to the honey bait (7.36 ± 0.21 min) than to the tuna (4.49 ± 0.11) (*χ*^2^ = 15.50, *df* = 1, *P* <  0.001).

The interaction effects of morphospecies × bait type (*χ*^2^ = 42.17, *df* = 4, *P* <  0.001) and morphospecies × vegetation type (*χ*^2^ = 183.7, *df* = 4, *P* <  0.001), were significant. Thus, post hoc mean contrasts showed that the foraging duration of *M.  minimum* increases from 1.25 ± 0.25 min in the oak forest to 47.33 ± 11.66 min in the agricultural land (*P* = 0.001). However, *Formica* spp. showed an inverse pattern with a foraging duration of 3.46 ± 0.59 min in the agricultural land increasing to 5.01 ± 1.35 min in the oak forest, which was not significantly different (*P* = 1.0). Foraging duration of *M. mexicana*, *Lasius* sp. (only found in agricultural land) ranged from 3 to 5 min, and they were not statistically different (*P* > 0.05). The foraging duration of *Pheidole* sp.1 was similar in both vegetation types (ranging from 2–4.2 min) (*P* = 0.99). The foraging duration of *Temnothorax* sp*.* was similar in both vegetation types (ranging from 3–7.0 min, *P* = 1.00). In relation to the morphospecies × bait type interaction, post hoc mean contrasts showed that the foraging duration of *M. minimum* on tuna bait (21.14 ± 10.25) was longer than on honey bait (1.0 ± 0 *P* < 0.001). While *Formica* spp. had longer foraging duration on honey bait (8.02 ± 1.93) than on tuna bait (2.49 ± 0.63). Also, the foraging duration of *M. minimum* on tuna bait was longer than *Formica* spp. or *Themnotorax* sp. (*P* < 0.001). The interaction effect of vegetation type × morphospecies × bait type (*χ*^2^ = 0.640, *df* = 2, *P* = 0.726) was not significant.

### Does land-use change modify the competitive hierarchy in temperate forest ant communities?

The estimation of the average DI of each morphospecies (based on the DI calculated for the baits exposed for 1 h, 2 h, and 4 h) showed values lower than 0.5, which indicates a generally submissive behavior, except for more dominant species such as *M. minimum* (Table 2). The GLMM model showed that vegetation type, morphospecies, bait type and bait exposure-time did not have a significant effect on numerical dominance (vegetation type, *χ*^2^ = 0.159, *df* = 1, *P* = 0.689; morphospecies, *χ*^2^ = 3.647, *df* = 7, *P* = 0.819; bait type, *χ*^2^ = 0.849, *df* = 1, *P* = 0.344; bait exposure time, *χ*^2^ = 0.427, *df* = 2, *P* = 0.152). The effects of vegetation type × morphospecies (*χ*^2^ = 2.466, *df* = 4, *P* = 0.650), vegetation type × bait type (*χ*^2^ = 0.013, *df* = 1, *P* = 0.907), vegetation type × bait exposure-time (*χ*^2^ = 0.371, *df* = 2, *P* = 0.942), morphospecies × bait type (*χ*^2^ = 1.368, *df* = 6, *P* = 0.967), morphospecies × bait exposure-time (*χ*^2^ = 1.368, *df* = 14, *P* = 0.967), bait type × bait exposure-time (*χ*^2^ = 0.451, *df* = 2, *P* = 0.911), and the vegetation type × morphospecies × bait type interaction × bait exposure-time (*χ*^2^ = 1.112, *df* = 84, *P* = 0.439) were not significant.

**Table 2:** Average (median ± SE, *n*) Numerical Dominance Index (DI) by ant morphospecies at the three time points of exposure in the Petri dishes (1 h, 2 h and 4 h).

Morphospecies	Time of exposure			
	1 h	2 h	4 h	Average DI
*Formica* spp.	0.01 ± 0.01, 6	0.07 ± 0.03, 5	0.06 ± 0.02, 6	0.05 ± 0.01^a^, 6
*Lasius* sp.	0, 1	–	0 ± 0, 2	0 ± 0, 2
*C. picipes pilosulus*	–	–	0, 1	0, 1
*M. minimum*	0.25 ± 0.25, 4	0.60 ± 0.24, 5	0.27 ± 0.12, 6	0.35 ± 0.14^a^, 6
*Pheidole* sp.1	0.05 ± 0.05, 6	0 ± 0, 2	0.23 ± 0, 5	0.17 ± 0.07^a^, 6
*Pheidole* sp.2	–	–	0 ± 0, 2	0 ± 0, 2
*Temnothorax* sp.	0 ± 0, 4	0 ± 0, 5	0.01 ± 0.01, 6	0.00 ± 0.00^b^, 6
*M. mexicana*	0 ± 0, 3	0 ± 0, 2	0, 1	0 ± 0^b^, 4

(–) No individuals of this morphospecies registered. Different letters (a, b) indicate there were significant differences in the average DI between morphospecies (only where a sufficient number of individuals were registered for the morphospecies). Tukey Test *P* < 0.05.

*Monomorium minimum* was much more behaviorally dominant than all other species (Table 2) and presented mass recruitment (on average 51 ants per Petri dish). However, its incidence was intermediate (presence in 39 Petri dishes). Although the dominance values for *Formica* spp. and *Pheidole* sp. 1 were not significantly different from those of *M. minimum*, these species differ in their recruitment type and incidences (presence in Petri dishes). *Formica* spp. showed group recruitment (on average three ants per Petri dish) and was the morphospecies with the highest incidence (presence in 193 Petri dishes). In the case of *Pheidole* sp.1, it had group recruitment (on average 11 individuals per Petri dish), and its incidence was intermediate (presence in 33 Petri dishes). *Temnothorax* sp. and *M. mexicana* comprised the next group in the dominance hierarchy*.* In the case of *Temnothorax* sp., it had group recruitment (on average 3.7 ants per Petri dish), and its incidence was intermediate (presence in 71 Petri dishes). *Myrmica mexicana* showed group recruitment (on average two ants per Petri dish) and had a very low incidence (presence in seven Petri dishes). Finally, *Lasius* sp., *Pheidole* sp. 2 and *C. picipes pilosulus* formed a group consisting of submissive and rarely registered species. In the case of *Lasius* sp., it had group recruitment (on average one ant per Petri dish), and its incidence was very low (presence in three Petri dishes). *Pheidole* sp.2 presented mass recruitment (on average 79 individuals per Petri dish) with a very low incidence (presence in three Petri dishes). Finally, only three individuals were registered for *C. picipes pilosulus*; presented group recruitment and a very low incidence (presence in one Petri dish).

### Does change in land use alter foraging strategies, favoring one species over another (Discovery-dominance trade-off)?

**Figure 1:**
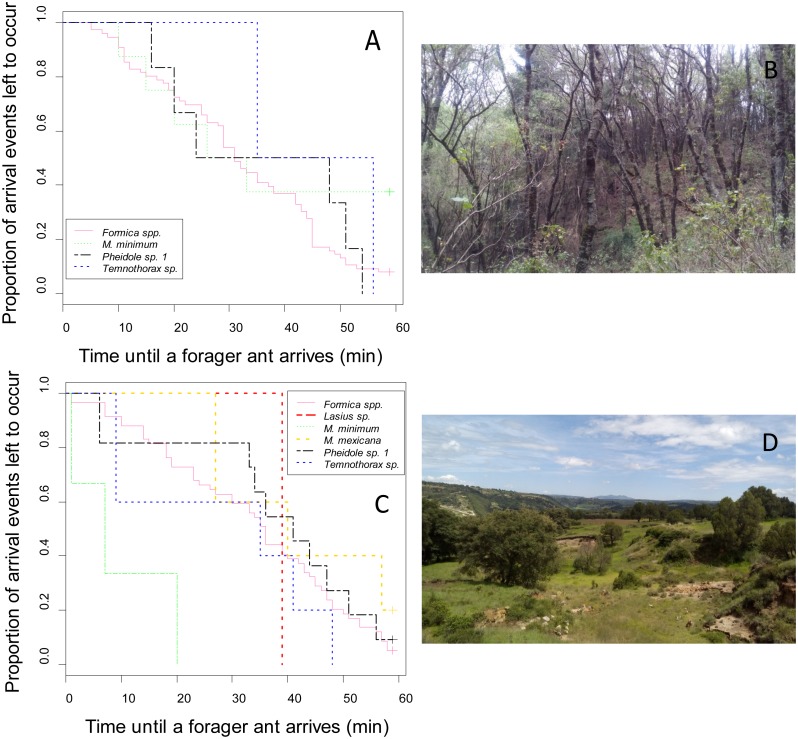
Ant morphospecies’ probability of visiting baits for foraging during the 60-min observation periods. The *y*-axis represents the likelihood of arrival events still occurring. The *x*-axis, is the time until an ant forager arrives from the start of the observation period. The analysis only includes ant species that arrived at the baits in both (A, B) oak forest and (C, D) agricultural land. Photos by Carlos Lara.

The bait exposure-time did not have a significant effect on numerical dominance (see results of the GLMM model of Dominance hierarchy). Considering the arrival times during the one hour observation, neither the type of vegetation (*χ*^2^ = 0, *df* = 1, *P* = 0.854) nor the type of bait (*χ*^2^ = 0.3, *df* = 1, *P* = 0.584) had a significant effect on the arrival times of the ants to the resources offered in the baits. However, vegetation type changed the different ant species probability of visiting baits exposed during 1 h. In the oak forest (Fig. 1B), the arrival times of *Formica* spp., *Temnothorax* sp. and *M. minimum* were continual during that hour; while *Temnothorax* sp. was the last ant species to reach the baits (Fig. 1A). However, in the oak forest (Fig. 1A), there were no significant differences in the arrival times of these morphospecies (*χ*^2^ = 2.8, *df* = 3, *P* = 0.432). In the agricultural land (Fig. 1D), the ant’s arrival times to the resources offered in the baits differ significantly (*χ*^2^ = 17, *df* = 5, *P* = 0.004). *M. minimum* (which also had the highest DI values) was the first ant species to reach the baits (Fig. 1C), and its arrival time differed significantly of the other ants (*χ*^2^ = 12.6, *df* = 1, *P* = 0.0004). The arrival times of the rest of the ants were similar (*χ*^2^ > 1.2, *df* = 1, *P* > 0.267). The last ant species to reach the baits was *Lasius* sp., although only one event was recorded for this ant.

## Discussion

In this study, we found that the eight registered morphospecies behaved as subdominant (*M. minimum*, *Pheidole* sp. 1 and *Formica* spp.) or subordinate species (*Temnothorax* sp. and *M. mexicana*). None of the observed species seem to fit the profile of dominant, which agrees with what was predicted for this type of communities where lower ant dominance is expected. Of the two baits, more visits were recorded to the tuna. In morphospecies such as *M. minimum, Pheidole* sp.1, and *Pheidole* sp.2, the higher number of visits to this type of bait could indicate a preference for protein resources or a limitation of that resource. In the case of *M. minimum*, it also spent more time foraging on tuna bait. The transformation of the oak forest does not seem to have much effect on dominance (dominance hierarchy), but it does affect the morphospecies’ foraging times (*M. minimum* had greater foraging times in the agricultural land) and arrival times (in the agricultural land, *M. minimum* was the first ant to arrive to the baits). Also, this change in land use resulted in a greater abundance of ants of all registered species. In addition, three species that were present in the modified habitat (agricultural land) were not present in the original habitat, *Pheidole* sp.2, *Lasius* sp., and *C. picipes pilosulus*. We found little evidence of the discovery-dominance trade-off and instead found diversity in the strategies used by the different species to access resources. This diversity can be seen in the foraging strategies of the generalized Myrmicinae *M. minimum*, the cold-climate specialist *Formica* spp. and rare ants such as the cold climate specialist *Lasius* sp.

### Differences between the two vegetation types

A low number of species were registered in this study, which agrees with the low richness (25 morphospecies) reported for this site (Silva Rodríguez, 2015). Of the eight morphospecies recorded, five were found in both vegetation types (*M. minimum*, *Formica* spp., *Pheidole* sp.1, *Temnothorax* sp., and *M. mexicana*) and only three were found exclusively in the agricultural land (*Pheidole* sp.2, *Lasius* sp. and *C. picipes pilosulus*). The type of vegetation seemed to have little influence on the hierarchy of dominance, but it did affect the arrival and foraging times of the morphospecies, and especially the number of visits to the baits. Both for the ant community as a whole (across species) and for individual morphospecies (within species), the total number of visits was higher in the agricultural land. The greater number of visits recorded in the agricultural land may be due to the fact that in this type of open vegetation, the temperature and solar radiation are usually higher (Barranco-León et al., 2016), which, for physiological reasons, may increase the activity of the ants (Retana & Cerdá, 2000). The most abundant morphospecies differed according to the vegetation type. In the oak forest, the cold-climate specialists *Formica* spp. and *Temnothorax* sp. were more frequently recorded at the baits. While, in the agricultural land, the generalized Myrmicinae *M. minimum* was more abundant and had longer foraging times. This coincides with what we expected as *Formica* spp. and *Temnothorax* sp. are cold-climate specialists, which are better adapted for the cooler conditions of the oak forest (Andersen, 1997; Andersen, 2000; Cuautle, Vergara & Badano, 2016). In the case of the *Formica* genus, different studies point to it being a possible bioindicator in temperate ecosystems (Ellison, 2012) and species of this genus are identified as key species around which temperate communities are structured (Trigos Peral et al., 2016). On the other hand, generalized Myrmicinae, such as *M. minimum*, are successful competitors that predominate under moderate levels of stress and disturbance but are not as active and aggressive as dominant Dolichoderinae, which are aggressive species most abundant in environments with low levels of stress and disturbance (Andersen, 1997). Interestingly, as with our study site, Dominant Dolichoderines are absent in cool-temperate regions elsewhere in the world. Behavioral dominance is usually exhibited by cold-climate specialists in these regions.

### Food preferences

The least submissive species (with the highest DIs) are generalized *Myrmicinae* (i.e., *Monomorium minimum, Pheidole* sp. 1). These species showed a preference for certain foods, which can be interpreted as a preference for less abundant (or scarce) food sources, in this case, proteins. This suggests that more dominant ants or generalists are able to monopolize the resources that are more desirable because of their nutritional quality or scarcity. This finding contrasts with other studies in temperate ecosystems where the food preference, especially of the dominant species, is for carbohydrates (Houadria et al., 2015). *Formica* spp. had a longer foraging duration on honey bait, while, *Temnothorax* sp. and *M. mexicana*, used both types of resources, possibly to reduce competition with other ants in the community for protein sources. This inference is also supported by the longer foraging times registered to tuna bait, in the agricultural land by *M. Monomorium*, a generalized Myrmicinae.

### Dominance hierarchy

When analyzing the hierarchy of dominance in the community (measured with the numerical dominance index), we found that the ants had very low indexes (<0.5) indicating subdominant or submissive behavior. *M. minimum* was the species that obtained the highest recruitment value and DI, but its incidence was intermediate. Meanwhile, *Formica* spp., submissive and with group recruitment, had the highest incidence. This tells us that *Formica* spp. is important in the structure of the ant community, as it appears to be having an impact on a more extensive area. Although foraging in smaller groups, its larger range allows it to reach a greater number of food resources.

These results seem to confirm what is anticipated for this type of communities. In temperate ecosystems, there are fewer dominant species than, for example, in the tropical (e.g., García-Martínez et al., 2015) or arid and semi-arid (seasonal) ecosystems of Australia (Arnan, Gaucherei & Andersen, 2011) where the richness and abundance of dominant species are high. Fewer dominant species promotes low competition, which is then reflected in a lower number of registered species and low dominance indices. However, the inclusion of the different species recorded in this study in one of the two categories and subcategories indicated by Trigos Peral et al. (2016), or one of the three categories proposed by Cerdá, Arnan & Retana, (2013), is not so simple. The absence of a dominant element is evident, which also agrees with what is expected for temperate ecosystems. In stressful environments such as oak forests with low temperatures, the absence of dominant Dolichoderinae is anticipated, and species adapted to these stressful environments assume dominance (Andersen, 2000; Andersen, 1997; Cuautle, Vergara & Badano, 2016), which in this ant community are the cold-climate specialists such as *Formica* spp. and *Temnothorax* sp. In disturbed environments, such as agricultural land, the absence of dominant Dolichoderinae and the predominance of the generalized Myrmicinae are also expected (Andersen, 2000; Arnan, Gaucherei & Andersen, 2011), in the case of our study *M. minimum.* Land use change from forest to agricultural land will cause a change in microclimatic conditions as temperature, and it is expected to find colder conditions in the oak forest than in the open vegetation (Cuautle, Vergara & Badano, 2016; Barranco-León et al., 2016). In this study, the differences in microclimatic conditions (especially temperature) between habitats, might explain the predominance of different functional groups in each type of vegetation.

### Discovery-dominance trade-off

The results of this study do not support the existence of a discovery-dominance trade-off in this temperate ant community. This is in agreement with a study by Parr & Gibb (2012), that found the discovery-dominance trade-off could be relaxed by the substantial effect temperature variance has on foraging activity and potentially by other trade-offs such as the trade-off between stress tolerance and competitive dominance (Bestelmeyer, 2000). As change in land use from forest to agricultural land is likely relaxing the discovery-dominance trade-off, the warmer ground temperatures of the disturbed vegetation types may be facilitating operation of other trade-offs such as stress tolerance and competitive dominance in the study site. The stress tolerance and competitive dominance trade-off hypothesis points out that dominants ants will forage when temperatures are optimal, but when temperatures are close to the physiological limits (critical temperatures) dominants will reduce their foraging activity, and this gives subordinate ants opportunity to forage in these extreme temperatures (Cerdá, Arnan & Retana, 2013). The opposite situation is also valid; ants tolerant to cold climatic (cryophilic) conditions can forage at lower temperatures than the dominant ants (Bestelmeyer, 2000). Parr & Gibb (2012) found that in structurally complex habitats such as forest, the increased heterogeneity and rugosity of the environment, can also relax these trade-offs as these factors affect discovery by slowing recruitment and altering species interactions, which prevent aggressive species from monopolizing resources.

### Differential behavioral strategies and dominance hierarchy of morphospecies

Although a discovery-dominance trade-off was not evident in the behavior of the ants in this study, differences in abundance, incidence (Petri dish occupation) and recruitment numbers (mean number of ants per Petri dish) were recorded. These differences could be indicating that the ants of this community may be using different strategies to access and dominate resources. As expected in temperate communities, this community has a simple dominance hierarchy (similar to that proposed by Trigos Peral et al., 2016), which appears to lack the territorial dominance level. On the top of this hierarchy would be *M. minimum,* which could be defined as a subdominant species (*sensu* Cerdá, Arnan & Retana, 2013). Not only was this ant the first to arrive but its prevalence at the 4 h time point demonstrates its dominance of the food resources. In the group of *M. minimum*, *Pheidole* sp.1 seems to be good at discovery, although the low number of this species registered at the midtime (2 h), could be indicating that it is not as good at maintaining and defending food resources. Below *M. minimum,* we could identify two groups of subordinate species, one formed by the cold-climate specialist, *Formica* spp. and *Themnotorax*, and the opportunist *M. mexicana*, and another group formed by the rare species (*Lasius* sp. *,C. picipes pilosulus*). The rare species, *Lasius* sp. and *C. picipes pilosulus*, would be in the subordinate category too, although their behavior would be better interpreted as that of an insinuator species (*sensu* Wilson, 1971). Insinuators arrive in small numbers and discretely steal food (*see* Yitbarek, Vandermeer & Perfecto, 2017). What about the cold-climate specialist *Formica* spp. and *Temnothorax* sp.? Could *Formica* spp. be a dominant ant? Its DI does not support this idea, but the number of Petri dish occupied by this ant shows that *Formica* spp. uses a high percentage of the available resources, which could be associated with its defense of the territory. It is well known that this genus is a dominant ant species in temperate forest (Savolainen & Vepsäläinen, 1989; Vepsäläinen & Pisarski, 1982). *Temnothorax* sp. was the second most prevalent ant in the Petri dishes, and its behavior was similar to that reported for *Formica* spp. We think that although *Formica* spp. and *Temnothorax* sp. were found in both vegetation types, they were probably part of the original ant community in the oak forest. The agricultural lands were a result of the modification of the native oak forest, so it is possible that *Formica* spp. and *Temnothorax* sp. migrated to the agricultural land; and that conversely, Myrmicinae could have moved to the oak forest from the agricultural land. The higher abundance of the cold-climate specialist in the oak forest may be a reminder of the previous history/land use. It is likely that the degree of disturbance in the temperate oak forest of the “La Malinche” is blurring the limits between the oak and agricultural land ant communities. The low number of morphospecies recorded in this study may also be due to a long history of disturbance in the zone.

Ants have been shown to be effective indicators of the ecological impact of human disturbance. As temperate forest clearing and land use change are accelerating across the globe, there is an urgent need for the study of the temperate ant communities especially in transition zones between temperate and tropical ecosystems, where Nearctic and tropical faunal elements such as ant communities are in contact with each other. The present study corroborates the proposal/theory that dominance hierarchies are simpler in temperate ecosystems than in tropical ecosystems. However, there is a need for a hierarchy categorization that is consistent both in its terms and application; one that may be used for all kinds of ecosystems. In this particular temperate ecosystem, the top dominant category appears to be absent; the subdominant category is well represented by *M. minimum,* a generalized Myrmicinae, and *Pheidole* spp. and insinuator species such as *Lasius* sp. and *C. picipes pilosulus* represent the subordinate category. *Formica* spp. is in the middle of these two categories, with no clear categorization as subdominant or subordinate species.

##  Supplemental Information

10.7717/peerj.6255/supp-1 Data S1 Raw data of Survival Analysis (”time failure analysis”) used to analyze differences in ant arrival times to either bait or vegetation type The number of ants of different species to visit baits exposed during 1 h.

## Additional Information and Declarations

### Field Study Permissions

The following information was supplied relating to field study approvals (i.e., approving body and any reference numbers): The field research reported here was performed using the required permit (SEMARNAT No. DGVS/06901/15).

### Data Availability

The following information was supplied regarding data availability: The raw data are provided in the Supplemental Files.
